# Don't Have a Doubt, Get the Catheter Out: A Nurse-Driven CAUTI Prevention Protocol

**DOI:** 10.1097/pq9.0000000000000183

**Published:** 2019-05-24

**Authors:** Melissa M. Schiessler, Lisa M. Darwin, Amber R. Phipps, Lindsay R. Hegemann, Brenda S. Heybrock, Andrew J. Macfadyen

**Affiliations:** From the Performance Improvement, Pediatric Intensive Care Unit, and Infection Prevention Departments, Children’s Hospital & Medical Center, Omaha, Neb.

## Abstract

Despite the use of sterile technique for indwelling urinary catheter insertion, as well as use of the defined catheter-associated urinary tract infection (CAUTI) bundle elements per Children’s Hospitals’ Solutions for Patient Safety, the CAUTI rate in the pediatric intensive care unit (PICU) at a free-standing pediatric hospital was increasing. In 2017, the PICU accounted for 87% of the organization’s CAUTIs and 65% of the total indwelling catheter device days. With an important risk factor for CAUTIs being the duration of catheterization, the indication for catheters became an organizational executive priority.

**Methods:**

An early 2017 review of the bundle elements identified that the indication for catheterization was not consistently addressed in daily patient rounds. A multidisciplinary project team applying the Plan, Do, Check, Act methodology developed an evidenced-based, nurse-driven indwelling urinary catheter removal protocol. This protocol allows nursing autonomy when removing a catheter by providing clinical indications for catheter use and promoting prompt removal when no longer indicated.

**Results:**

Indwelling urinary catheter device days in the PICU decreased by 28% within 6 months of protocol implementation. The PICU CAUTI rate declined from 4.8 (per 1,000 device days) in 2017 to 0.8 in 2018, 1 year after protocol implementation.

**Conclusions:**

Providing the bedside nurse with an evidence-based protocol that is driven by specific patient indications and diagnoses allows them to practice autonomously in catheter removal. Prompt removal of indwelling urinary catheters results in decreased device days and decreased incidence of CAUTIs.

## INTRODUCTION

The catheter-associated urinary tract infection (CAUTI) rate in a 27-bed pediatric intensive care unit (PICU) was becoming an increasing problem. In 2016, the rate of CAUTIs for all inpatient units at the organization measured 1.25 (number of infections per 1,000 device days), and in the PICU, the rate measured 1.53. By September 2017, the PICU had already identified 5 CAUTIs among their patients, at a rate of 18.7, whereas the rest of the inpatient units had zero.

CAUTIs have a significant impact on morbidity, mortality, and healthcare expenditure, and yet to a great extent, they are preventable.^1^ The Children’s Hospitals’ Solutions for Patient Safety, a network of Children’s Hospitals with a collaborative effort to transform pediatric patient safety, indicate an estimated cost of $1,000 per CAUTI.^2^ The complication of a urinary tract infection (UTI) can increase a patient’s hospital average length of stay by 0.4 days for an asymptomatic UTI and 2.0 days for a symptomatic UTI.^3^ Other complications associated with the use of indwelling urinary catheters include urethritis, urethral strictures, hematuria, bladder perforation, catheter obstruction, and urosepsis.^3^ With the increase in the length of stay due to a CAUTI and the Centers for Medicare and Medicaid Services prohibiting payment for the additional cost of services resulting from preventable hospital-acquired conditions (HAC),^4^ there was a clear need to decrease the incidence of CAUTIs in the PICU.

## BACKGROUND

A PRISM (Pediatric Risk of Mortality) III Severity of Illness index by Virtual Pediatric Systems, LLC (Los Angeles, CA), which is a physiologic-based risk of mortality tool used to predict the intensive care outcomes of children less than 18 years of age, identified the PICU as having a risk of mortality of 3.82% in 2017. From a statistical standpoint, this is significantly higher than the Virtual Pediatric Systems reference group of 2.00% (*P* < 0.0001).^5^ With the unit comprised a mixed cardiac and medical/surgical patient population with a high level of acuity, CAUTI prevention became an executive priority.

Criteria for defining CAUTIs at the organization come from Solutions for Patient Safety and the National Healthcare Safety Network. The unit was already utilizing a sterile technique for indwelling urinary catheter insertion, as well as defined CAUTI bundle elements. A focus became reviewing the bundle elements for indwelling urinary catheters to assure they were appropriately followed. In doing so, the unit CAUTI champion, a bedside and charge nurse, identified an inconsistency in the daily discussion of an indication for the indwelling urinary catheter. Multiple studies show that between 21% and 55.7% of urinary catheters are placed in patients who do not have an appropriate indication and, therefore, may not even need a catheter.^6^ The catheter indication necessity became the aim of the project as a means to decrease urinary catheter device days and ultimately decrease unit CAUTIs.

## METHODS

A literature review was performed utilizing the keywords PICU, urinary catheters, and nurses. Consistent themes identified in the literature to decrease urinary catheter device days were the involvement of nurses in designing interventions to improve patient care and continuing to grant nurses responsibility in sustaining the improvement.^7^ With the unit CAUTI champion and a project manager as leaders, a multidisciplinary team consisting of the PICU Medical Director, Infection Prevention nurses, an OR manager, a Neonatal Nurse Practitioner, an analyst, and PICU nursing leadership was established.

We applied the Plan, Do, Check, Act (PDCA) methodology in protocol development. The first step included determining the appropriate time for indwelling catheter review. Twenty-four hours was defined as the time that nursing staff would begin questioning catheter necessity. The second phase of protocol development was distinguishing indications for indwelling catheter necessity. Attending physicians and clinical service chiefs were utilized to identify special considerations for urinary catheter indications and to promote future buy-in of the protocol. Special considerations include consulting with Urology and Pediatric Surgery before removal of a catheter placed by the service. These specialty services commonly order and maintain indwelling urinary catheters for their patients for extended periods due to a urological malformation or specific postoperative consideration with the need for catheter reinsertion having the potential to cause great harm. Another special consideration was consulting a Certified Wound, Ostomy and Continence Nurse to discuss catheter necessity if a pressure ulcer or wound is present around the coccyx or perineal area.

**Fig. 1.**
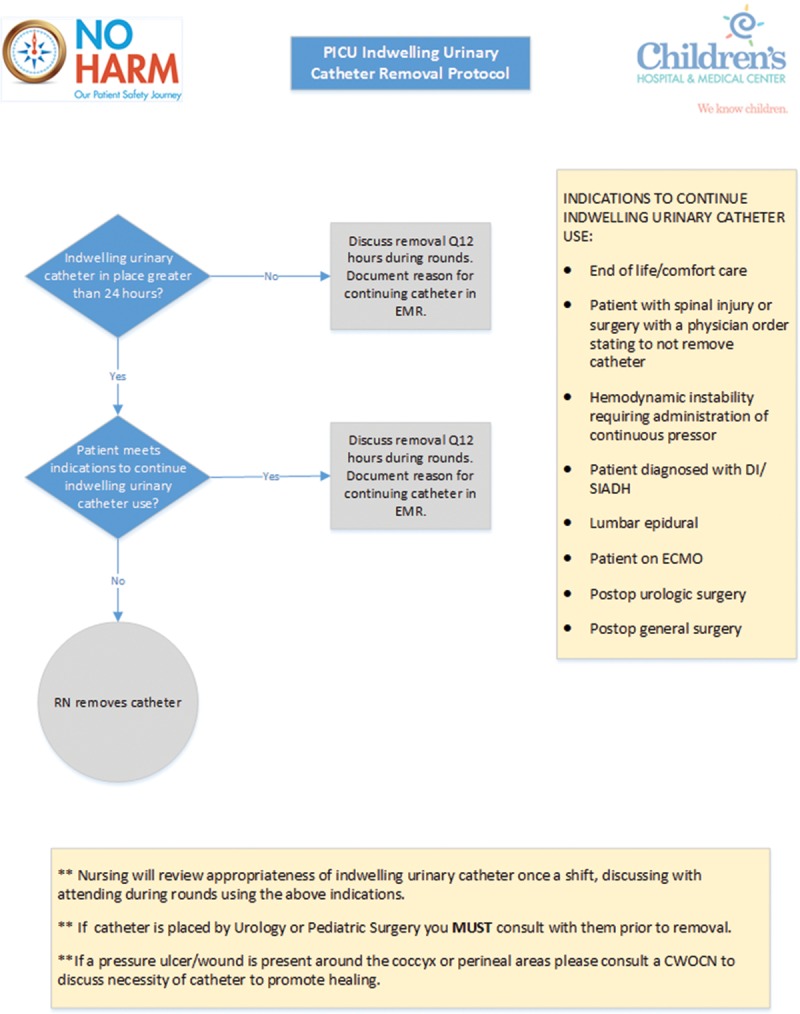
PICU indwelling urinary catheter removal protocol. Nurse-driven protocol formulated from evidence-based research utilized to promote prompt decision making and discontinuation of indwelling urinary catheters through indication guidance. CWOCN indicates Certified Wound, Ostomy and Continence Nurse. RN, nurse; EMR, electronic medical record; SIADH, syndrome of inappropriate antidiuretic hormone secretion; DI, diabetes insipidus.

Due to the inconstancy of discussion for catheter necessity each shift, a location in the electronic medical record was then built for the frontline staff to document the approved indications for use. This addition served as both a reminder and a tracking mechanism for necessity discussion. Education for attending physicians and bedside nursing staff began in October 2017, with presentations and explanations of the protocol and its importance in CAUTI prevention. The unit CAUTI champion was available as a direct resource at the bedside for staff and was able to field questions and comments. Starting in November of 2017, the protocol was put into practice (Fig. 1). The number of device days, as calculated by the organization’s Infection Preventionist, was identified as the metric for assessing protocol impact. Activities were designed solely for evaluation of processes and quality improvement and did not require Institutional Review Board (IRB) approval.

## RESULTS

**Fig. 2.**
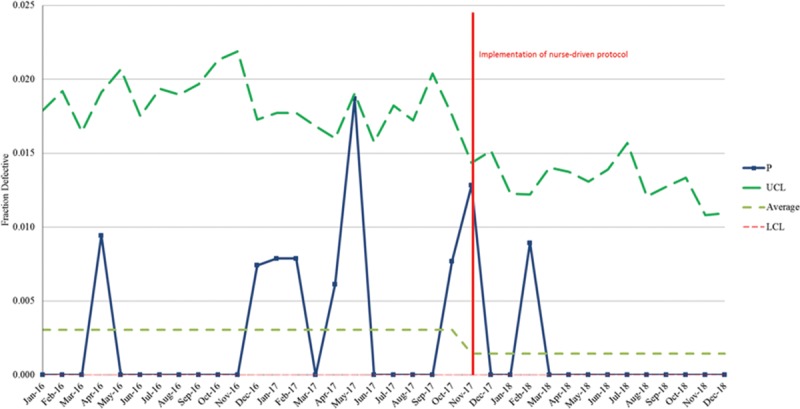
PICU catheter-associated urinary tract infections. Decrease in actual catheter-associated urinary tract infections following the nurse-driven protocol implementation when compared with PICU indwelling urinary catheter device days. UCL, upper control limit; LCL, lower control limit.

**Fig. 3.**
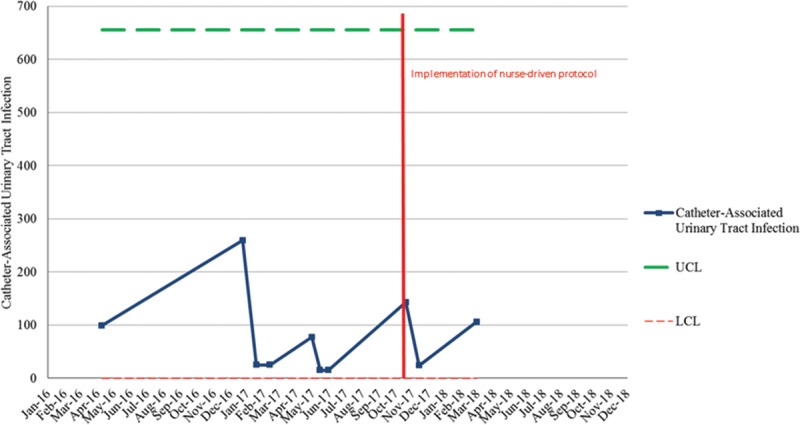
Time between catheter-associated urinary tract infection events. Increase in the time between catheter-associated urinary tract infections since implementation of nurse-driven protocol. UCL, upper control limit; LCL, lower control limit.

One month after implementation of the protocol, the unit had decreased their device days by 60%. In that same month, there was a CAUTI identified in a patient. The CAUTI champion performed a retrospective chart review and completed a thorough cause analysis of the CAUTI event. It was identified that the provider had several concerns resulting in the catheter not being removed. These concerns included development of rhabdomyolysis and elevated patient laboratories, resulting in the need for accurate and continuous intake and output measurement through use of the indwelling urinary catheter. This infection was used as a “live and learn” moment to educate the staff further and provide open discussion for any future diagnoses that could have the potential to be added as indicators for catheter use. The next month the unit continued to decrease the number of device days and subsequently had zero identified CAUTIs. One year following the protocol implementation, the PICU continues to have only 1 CAUTI (Fig. 2) and has increased the duration of time between CAUTI events (Fig. 3). With the utilization of the nurse-driven protocol to promote alignment with bundle compliance, device days in the PICU have shown a measurable decrease.

One indication that has affected increases in device days following protocol initiation was the use of extracorporeal membrane oxygenation (ECMO) in the PICU during January, February, and November of 2018. Assessment of patients with an indwelling urinary catheter in January and February 2018 found that 10 patients were on ECMO for a combined duration greater than 1,100 hours. Once a patient is placed on ECMO, the staff is unable to insert or remove any invasive monitoring device due to the high risk of bleeding. Therefore, indwelling urinary catheters must remain in place, while a patient is on ECMO.

As part of the PDCA methodology, the team continues to reevaluate the protocol and indications for urinary catheterization as questions from providers arise. The PICU CAUTI champion will retrospectively review every CAUTI to identify indications of catheter presence and provide staff with educational reminders on protocol utilization. The information gathered will lead to the PICU multidisciplinary HAC Prevention Workgroup review that is done following all CAUTIs.

## DISCUSSION

UTIs are directly associated with the use of indwelling urinary catheters.^3^ Although the reduction of HACs remains a national patient safety goal, implementing strategies at the bedside to reduce both the risk of infection and indwelling urinary catheter device days for hospitalized patients provides bedside nursing staff with the tools they need to protect their patients. The development of a protocol helped this unit better align with the organization’s strategies of providing safe, high-quality care and delivering an outstanding patient and family experience. It also allowed for the empowerment of the bedside nursing staff, development of critical thinking skills, and for reminders of risk reduction daily.

Following the positive results of the study, other inpatient units within the organization are being identified for the development of their own nurse-driven indwelling urinary catheter removal protocol based on their patient population and evidence-based research. In particular, a step-down unit has implemented a similar protocol which has also resulted in a decrease of indwelling urinary catheter device days. Ongoing analysis and PDCAs are still being employed to identify diagnoses or conditions that may also be an indication for catheter use.

In conclusion, although aseptic insertion of indwelling urinary catheters and evidence-based care bundle utilization are important in the reduction of CAUTIs, they are not enough to completely prevent them. Nursing autonomy with patients and situational awareness promoted through catheter necessity discussions are keys in delivering timely care. A nurse-driven indwelling urinary catheter removal protocol has proven to reduce CAUTIs and the length of catheter days in this PICU.

## ACKNOWLEDGMENTS

Amber Marquiss, Analytics Supervisor, Performance Improvement, assisted the study. VPS data were provided by Virtual Pediatric Systems (VPS), LLC. No endorsement or editorial restriction of the interpretation of these data or opinions of the authors has been implied or stated.

## DISCLOSURE

The authors have no financial interest to declare in relation to the content of this article.
